# A realistic approach for evaluating antimicrobial surfaces for dry surface exposure scenarios

**DOI:** 10.1128/aem.01150-24

**Published:** 2024-10-04

**Authors:** Yi Enn Cheong, Ralph Weyandt, Wilma Dewald, Timo Tolksdorf, Laura Müller, Armin Braun

**Affiliations:** 1 Volkswagen AG, Group Innovation, Wolfsburg, Germany; 2 Bioservices Department, SGS Institut Fresenius GmbH, Taunusstein, Germany; 3 Preclinical Pharmacology and Toxicology, Fraunhofer Institute for Toxicology and Experimental Medicine – Hannover (Germany), Member of the German Center for Lung Research (DZL), Biomedical Research in Endstage and Obstructive Lung Disease Hannover (BREATH) research network Hannover (Germany), Member of the Fraunhofer Excellence Cluster of Immune Mediated Diseases (CIMD) and Institute of Immunology, Medizinische Hochschule Hannover, Hannover, Germany; Centers for Disease Control and Prevention, Atlanta, Georgia, USA

**Keywords:** fomite-transmitted infection, antimicrobial coating, surface coating, antimicrobial activity, efficacy testing, antimicrobial, antibacterial, antiviral agents, realistic condition, real-life scenarios

## Abstract

The severe acute respiratory syndrome coronavirus 2 pandemic has raised public awareness about the importance of hygiene, leading to an increased demand for antimicrobial surfaces to minimize microbial contamination on high-touch surfaces. This is particularly relevant in public and private transportation settings, where surfaces frequently touched by individuals pose a significant, yet preventable, risk of infection transmission. Typically, the antimicrobial activity of surfaces is tested using test methods of the International Standards Organization, American Society for Testing and Materials, or Japanese Industrial Standards, which involve complete submersion in liquid, elevated temperature (37°C), and prolonged (24 h) contact periods. However, these conditions do not accurately represent real-world scenarios where surfaces are exposed to air. In this study, we propose a modified test method designed to better reflect real-life conditions in the intended end-use setting. The modifications included using deionized water instead of nutrient broth while preparing bacterial inoculum, applying a small test inoculum to the surface and allowing it to dry, maintaining ambient temperature and relative humidity throughout the contact period, and reducing the contact period to 4 h. With this modified approach, the antimicrobial activity of 20 samples was reassessed. This screening revealed that out of 20 samples, only 2 samples were effective against all species, while 8 samples demonstrated partial effectiveness against selected species, and 10 samples showed no significant effect. These findings highlight the inadequacy of the current test standard and emphasize the urgent necessity for revised and adapted testing method to ensure a reliable and accurate evaluation.

**IMPORTANCE:**

The recent severe acute respiratory syndrome coronavirus 2 pandemic has sparked increased demand for antimicrobial surfaces to mitigate the risk of fomites-transmitted infection in both indoors and confined spaces. Commonly, the antimicrobial activity of these surfaces is assessed using test standards established by national standards bodies, which do not distinguish between different application scenarios. While these test standards are suitable for surfaces intended for submerged application, they are inappropriate for antimicrobial surfaces designed for dry surface exposure. The usage of these standards can lead to an overestimation of antimicrobial efficacy. Thus, this study introduces a modified dry exposure test method aimed at better reflecting real-life conditions in the intended end-use setting. Our results revealed the subpar antimicrobial performance of numerous samples, highlighting the necessity to revise and tailor the universal test standard to real-world scenarios in order to ensure a reliable and accurate evaluation.

## INTRODUCTION

Recently, the severe acute respiratory syndrome coronavirus 2 (SARS-CoV-2) global pandemic has raised significant awareness among the public on the importance of hygiene to reduce the spread of the viral infection. However, SARS-CoV-2 is only one example of a wide variety of airborne pathogens. Fomites remain a significant transmission route for various viruses and bacteria of high concern such as norovirus, poxvirus, *Pseudomonas aeruginosa*, *Escherichia coli*, and and so on (1, 2). Besides personal hygiene, car hygiene is also crucial since humans spend a substantial amount of time commuting in their own car or traveling as passengers in their daily lives. This confined and often shared space can potentially increase the risk of infection. In contrary to an open space, studies showed a threefold increment in the risk of transmission of respiratory infectious diseases within a confined space (3). While transmission generally occurs through physical contact between individuals, it is important to note that infectious particles can also be transferred from an infected person to inanimate surfaces, and subsequently to a susceptible host (4, 5). The massive ridership intensifies the transmission of microorganisms on public transportation. A study from Mexico revealed that the train and station surfaces are dominated by typical skin microbiota *Cutibacterium*, *Corynebacterium*, *Streptococcus*, and *Staphylococcus* (6). A recent investigation showed a mean microbial load of 273.7 CFU/cm^2^ on frequently touched surfaces in the commuter’s compartment in shared mobility (unpublished data, data not shown).

In recent decades, shared mobility services have gained significant popularity and are becoming more and more prevalent in cities all over the world. Instead of vehicle ownership, increasing population now relies on shared mobility such as ride-hailing, ride-sharing, and car-sharing that takes them to their destination (7). This mode of mobility has the potential to offer solutions to several environmental concerns and sustainability challenges, for instance, atmospheric pollution, occupied spaces, and so on (8). Prior to the SARS-CoV-2 outbreak, public transportation choices were predominantly influenced by travel time, convenience, and cost (9). However, the emergence of the pandemic shifted priorities toward minimizing infection risk, resulting in a substantial change in mobility patterns. This shift has led to a significant reduction in the usage of public transportation and shared mobility services (10–13).

In order to boost user’s confidence in shared mobility, original equipment manufacturers (OEMs) have been exploring and implementing technologies to tackle vehicle sanitation. Numerous researchers and companies are currently developing materials with antimicrobial functionalities to minimize the spread of infections (14). Generally, pathogenic microorganisms were killed by these antimicrobial materials via four mechanisms: (i) by actively releasing biocides (e.g., silver, zinc, copper ions, or nanoparticles), (ii) by contact-killing with immobilized organic molecules (e.g., quaternary ammonium compounds and chitosan), (iii) by photocatalytic or photodynamic action (e.g., TiO_2_ and photosensitizers), and (iv) by reducing adhesion of bacteria to surface (e.g., hydrophobic surface and micro/nano-structured surface) (15–18). The application of these antimicrobial technologies could assist in minimizing the risk of infection in vehicles’ interiors.

To evaluate the activity of these antimicrobial materials, appropriate standard test methods are necessary. Currently, the Japanese Industrial Standards (JIS) or International Standards Organization (ISO) 22196, 21702, 20743, and 18184 test standards are used by most manufacturers to assess antibacterial and antiviral activity on plastics and other non-porous surfaces, as well as textiles (19–22). These protocols share a similarity, where surfaces are tested using a liquid bacterial inoculum, which is cultivated at 37°C for 24 h. Viable bacteria are enumerated after 24 h and compared to counts on untreated surfaces (23–27). Another common standard used to determine the antimicrobial activity of immobilized antimicrobial agents under dynamic contact conditions is the American Society for Testing and Materials (ASTM) E2149 test method. This protocol involves immersion of test surfaces in liquid bacterial suspension with agitation for 1 h. However, both of these test methods are inappropriate because the testing parameters do not reflect dry surface exposure conditions (e.g., in vehicles or public transportation), and could result in overestimated antimicrobial activity and bacterial growth (23, 28, 29). To overcome this drawback, a number of researchers have reported several modified or alternative methods that better simulate these real-life conditions (23, 28–33). In order to address this issue, we developed a modified test method (based on ISO standard 22196 and ISO standard 21702) that reflects real-world exposure settings, by adjusting key parameters such as temperature, humidity, and contact time. The aim of this study was to evaluate the antimicrobial efficacy of 20 antimicrobial materials with our established test method.

## MATERIALS AND METHODS

### Preparation of test surfaces

Twenty antimicrobial surfaces were used in this study. Prior to use, each test surface (2 × 2 cm^2^) was decontaminated under a class-II biosafety cabinet (SterilGARDIII Advance, The Baker Company, Sanford, USA) using UV light for 15 min per side.

### Bacterial strains and growth conditions

*E. coli* ATCC 8739 (DSM 1576) and *Staphylococcus aureus* ATCC 6538 (DSM 799) were obtained from Leibniz Institute DSMZ-German Collection of Microorganisms and Cell Cultures GmbH, Germany.

Bacterial strains were stored at −80˚C on cryopreservation beads (Microbank; Pro-Lab Diagnostics, Merseyside, United Kingdom). Strains were sub-cultured on Plate-Count-Agar (Oxoid, ThermoFisher Scientific, PO5013A) two times prior to the experiment. Bacterial suspensions were prepared by inoculating single colonies from overnight culture into distilled water and adjusted to the appropriate cell concentration (~10^7^ CFU/mL) according to McFarland’s method (34).

### Antibacterial efficacy testing on textiles or porous and non-porous materials

About 50 µL of bacterial suspension was inoculated onto each test surface in the form of 5 µL per aliquot and left dried at 37°C for 30 min in the dark. Viable bacteria recovered from test surfaces were quantified at T0 (end of drying process) and at T4 (4 h contact time at 22.5 ± 2.5°C in the dark). For recovery, each test surface was neutralized in 5 mL phosphate-buffered saline enriched with N10 (30 g/L polysorbate 80, 30 g/L saponin, 3 g/L lecithine, 1 g/L histidine, and 5 g/L sodium thiosulfate) and vortexed vigorously before serially diluted in NaCl_phys_ (physiological water 0.85%, bioMérieux SA). About 1 mL of each dilution was mixed in duplicate with melted Tryptic Soy Agar (TSA) (Merck-Millipore 146429) and incubated at 37°C for 48 h. Plates with 30–300 colonies were quantified. Non-coated surface was used as control. All experiments are performed in triplicates.

### Viral strain and its culture conditions

Vaccinia virus strain Modified Vaccinia Virus Ankara (MVA) (ATCC VR-1508, ATCC, Manassas, Virginia, USA) was propagated in BHK-21 host cells (DSMZ ACC 61, Leibniz Institute DSMZ- German Collection of Microorganisms and Cell Cultures GmbH, Germany).

Host cells were cultivated in MEM-medium (Biowest, L0430-500) supplemented with 2 mM l-glutamine, 20% fetal calf serum (Biowest, S1810-500), and 1% antibiotic solution (Penicillin-Streptomycin, Biowest L0018).

For viral propagation, BHK-21 cells were infected with MVA and incubated at 37°C in the presence of 5% CO_2_, with >70% RH. Following cytopathic effects, the cells were subjected to a freeze/thaw cycle. Cell debris was removed via centrifugation at 120 × *g* for 10 min. Supernatant was collected and stored at −80°C until use.

Viral suspensions recovered from test surfaces were serially diluted and 0.1 mL of each dilution was titrated in 96-well Microtiter Microplate (Sarstedt AG, Nümbrecht, Germany) seeded with monolayers of BHK-21 cells. The plates were incubated at 37°C in the presence of 5% CO_2_, with >70% RH. Observation of cytopathic effect was performed by using an inverted microscope (Zeiss Televal 3, up to 400× magnification) after 6–7 days. The Tissue Culture Infectious Dose (TCID_50_/mL) was determined according to Spearman-Kärber analysis (35).

### Antiviral efficacy testing of textiles or porous materials and non-porous materials

About 50 µL of viral suspension was inoculated onto each test surface in the form of 5 µL per aliquot and left dried at 37°C for 30 min in the dark. Test surfaces were examined at T0 (end of drying process) and at T4 (4 h contact time at room temperature in the dark). Post drying, each sample was resuspended in iced MEM-medium depending on the material, by mechanical scraping (with cell scraper) and intense vortexing, prior to storage at −80°C until infectivity determination. Non-coated surface was used as control. All experiments are performed in triplicates.

## RESULTS AND DISCUSSION

**Fig 1:**
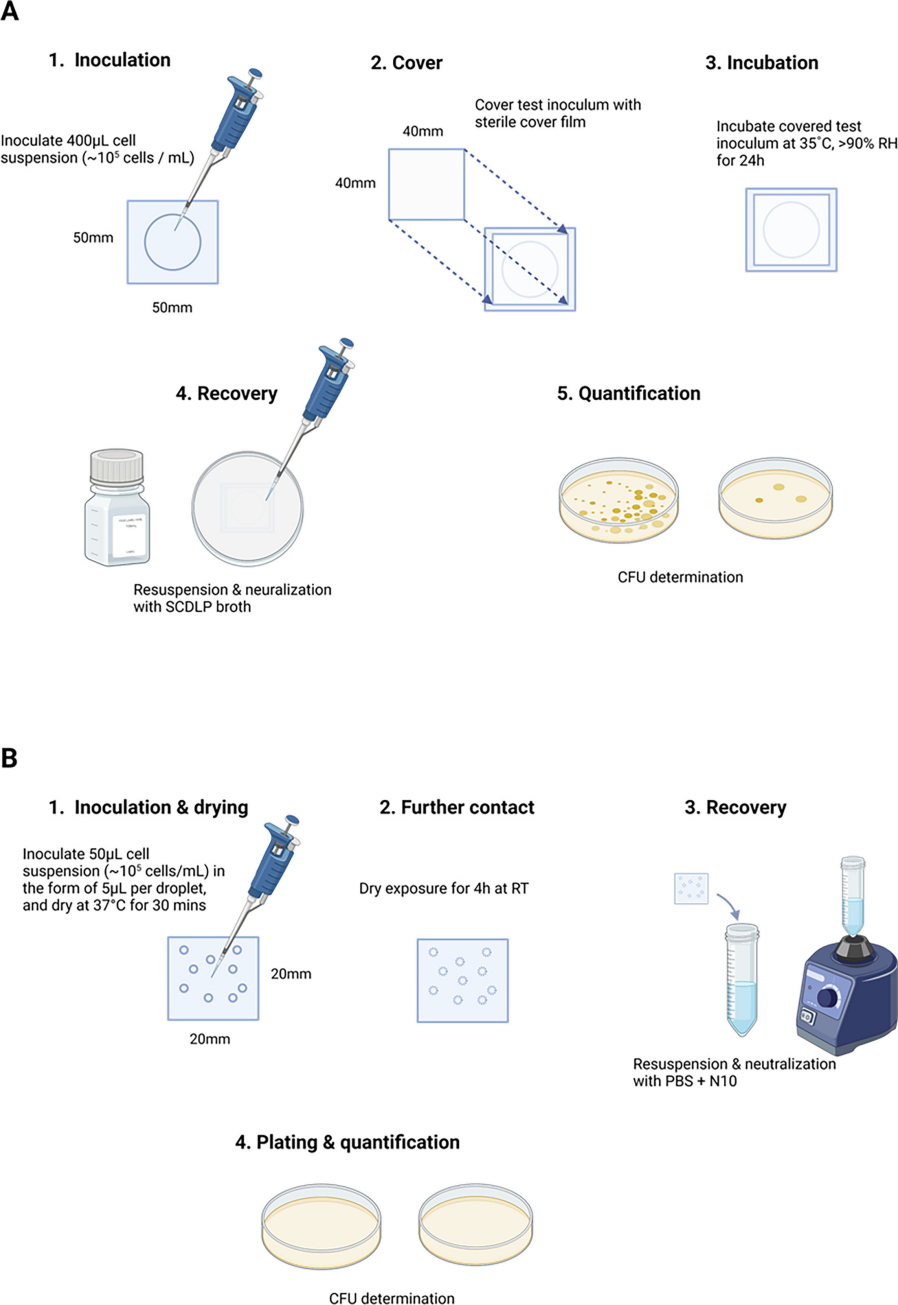
(A) Illustration of the standard ISO 22196 testing method. (B) Illustration of proposed modified dry testing method. Created with BioRender.com.

In this study, a test method was developed based on the ISO 22196 and 21702, with key modifications to simulate real-life dry exposure conditions and provide a more accurate evaluation on efficacy (Fig. 1). The adjusted parameters include (i) a small test inoculum (10 aliquots of 5 µL) was applied to the test item instead of the huge 400 µL inoculum volume; (ii) suspended test organisms are allowed to dry on the test surface simulating more realistic conditions rather than being covered with a film, resulting in a state of constant moisture; (iii) bacterial inoculum was prepared in distilled water instead of nutrient broth or saline solution; (iv) ambient temperature and relative humidity were applied during contact instead of 37°C; and (v) contact time was reduced from 24 to 4 h. With this method, a range of commercially available and developmental samples (formulation undisclosed due to proprietary reason) which have been reported as effective based on standard testing methods, were screened to re-evaluate their antimicrobial the efficacy and to aid in monitoring efficacy of new coatings under realistic dry exposure conditions (Table 1).

**TABLE 1:** Summary of antimicrobial test samples

Sample	Active ingredients	CAS no.	Commercial (C)/developmental (D) products	Concentration (wt%)	Test organisms	Test standard	Contact time	Reduction efficacy (log10)
A	Dimethyltetradecyl[3-(trimethoxysilyl)propyl]ammonium chloride	41591-87-1	C	1.5	Feline coronavirus	ISO 18184:2019	2 h	3.0
					Influenza virus	ISO 18184:2019	2 h	4.0
					Severe acute respiratory syndrome coronavirus 2 (SARS-Cov-2)	ISO 18184:2019	2 h	2.8
B	Dimethyloctadecyl[3-(trimethoxysilyl)propyl]ammoniumchloride	27668-52-6	C	1–3	Transmissible gastroenteritis virus (TGEV)	ISO 21702:2019	2 h	2.7
					*E. coli*	ISO 22196:2011	24 h	3.5
					*S. aureus*	ISO 22196:2011	24 h	3.5
C	Dimethyltetradecyl [3-(trimethoxysilyl)propyl]ammoniumchloride	41591-87-1	C	1.5–2	N/A	N/A	N/A	N/A
D	Alkyl(C12-16)dimethylbenzylammoniumchloride	68424-85-1	C	0.5	Modified vaccinia virus Ankara	EN14476:2013 + A2:2019	5 min	>4
					Feline coronavirus	EN14476:2013 + A2:2019	5 min	>4
					Murine hepatitis virus MHV-3	EN14476:2019	15 min	>4
					*P. aeruginosa*	PAS2424	24 h	>3
					*S. aureus*	PAS2424	24 h	>3
					*E. coli*	PAS2424	24 h	>3
					*Enterococcus hirae*	PAS2424	24 h	>3
					*C. albicans*	PAS2424	24 h	>3
E	Dimethyloctadecyl[3-(trimethoxysilyl)propyl]ammoniumchloride	27668-52-6	C	2.9	*Enterococcus hirae*	JIS Z 2801:2012	24 h	>4
					Methicillin-resistant *S. aureus* (MRSA)	JIS Z 2801:2012	24 h	>4
					*E. coli*	JIS Z 2801:2012	24 h	>4
					*K. pneumoniae*	JIS Z 2801:2012	24 h	>4
					*L. monocytogenes*	JIS Z 2801:2012	24 h	>4
					*Salmonella choleraesuis*	JIS Z 2801:2012	24 h	>4
					*C. difficile*	JIS Z 2801:2012	24 h	>4
					*P. aeruginosa*	JIS Z 2801:2012	24 h	>4
					Influenza virus H1N1	JIS Z 2801:2010	24 h	1.7
					Transmissible gastroenteritis virus (TGEV)	ISO 21702:2019	24 h	2.3
F	Zn pyrithione	13463-41-7	C	3	Feline coronavirus	ISO 18184:2019	24 h	2.5
G	ZnO nanoparticles	1314-13-2	D	N/A	Modified vaccinia virus Ankara	ISO 18184:2019	24 h	~3
					Severe acute respiratory syndrome coronavirus 2 (SARS-CoV-2)	ISO 18184:2019	24 h	1.0
H	ZnO	1314-13-2	D	N/A	*E. coli*	ISO 22196:2011	24 h	1.9
					*S. aureus*	ISO 22196:2011	24 h	3.6
I	ZnO	1314-13-2	D	N/A	*E. coli*	ISO 22196:2011	24 h	>4
					*S.aureus*	ISO 22196:2011	24 h	>4
J	Zn:DLC	N/A	D	N/A	N/A	N/A	N/A	N/A
K	AgCl	7883-90-6	C	0.4–0.5	Feline coronavirus	ISO 18184:2019	2 h	~1.5
L	Ag nanoparticles	7440-22-4	C		Murine hepatitis virus MHV-3	ISO 18184:2019	2 h	4.0
					Canine coronavirus	ISO 18184:2019	2 h	>4
M	Cu_2_O	1317-39-1	C	1.17	Human coronavirus (HCoV-229E)	ISO 18184:2019	2 h	>2
				1.17	Methicilin-resistant *S. aureus* (MRSA)	ATCCTM100-2019	2 h	4.0
				1.17	*K. pneumoniae*	ATCCTM100-2019	2 h	4.0
				1.17	*E. faecalis*	ATCCTM100-2019	2 h	4.0
				1.17	*C. auris*	ATCCTM100-2019	2 h	4.0
N	Untreated pine wood	N/A	D	N/A	N/A	N/A	N/A	N/A
O	Untreated birch wood	N/A	D	N/A	N/A	N/A	N/A	N/A
P	Cellulose	N/A	D	N/A	N/A	N/A	N/A	N/A
Q	Hydrophobic coated birch bark	N/A	D	N/A	N/A	N/A	N/A	N/A
R	Chitosan	9012-76-4	D	20	N/A	N/A	N/A	N/A
S	Chitosan	9012-76-4	D	20	N/A	N/A	N/A	N/A
T	Chitosan	9012-76-4	D	100	N/A	N/A	N/A	N/A

**Fig 2:**
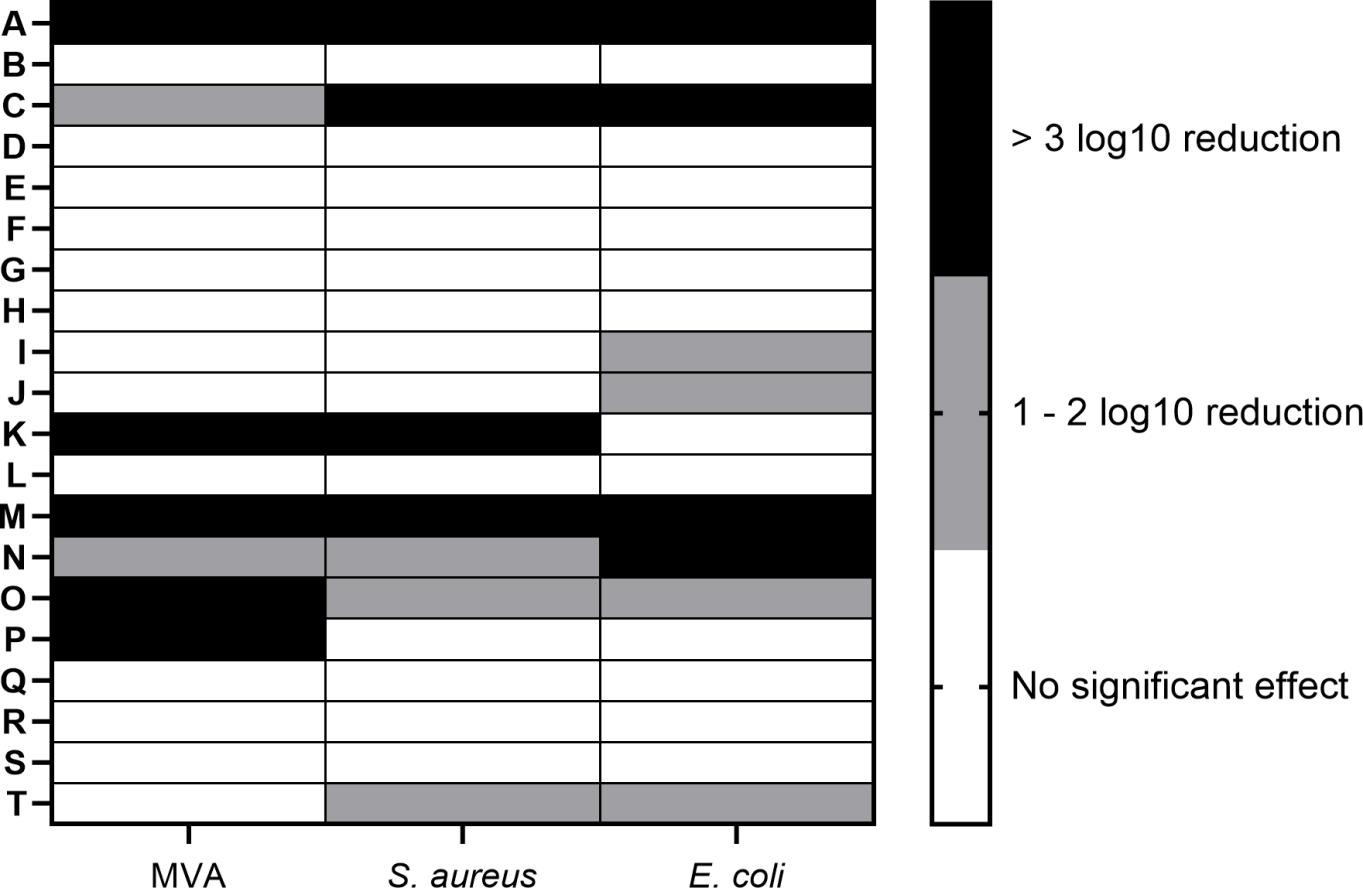
Efficacy of antimicrobial coatings against modified vaccinia virus Ankara, *S. aureus*, and *E. coli*. Data represent mean logarithmic reduction. (A–E) Quaternary ammonium compounds (QAC); (F–M) metal-based coatings; (N–T) bio-based samples.

In general, the results revealed a great variability in the antimicrobial activity among all test samples and all three target microorganisms, with efficacy ranging from less than 1 to greater than 3 log_10_ reduction. Efficacy testing was performed with approximately 5 × 10^5^ cells/mL using a 4.5-h contact time including drying. The antimicrobial activity of test surfaces against MVA, *S. aureus*, and *E. coli* is presented in Fig. 2. Out of 20 active ingredients, only 2 samples achieved at least 3 log_10_ reduction on all three species, 8 samples are partially effective (ranging from 1 to 3 log_10_ reduction) against selected species, and the remaining 10 samples showed no effect at all. Notably, even samples containing the same active substance showed variation in antimicrobial activity.

**Fig 3:**
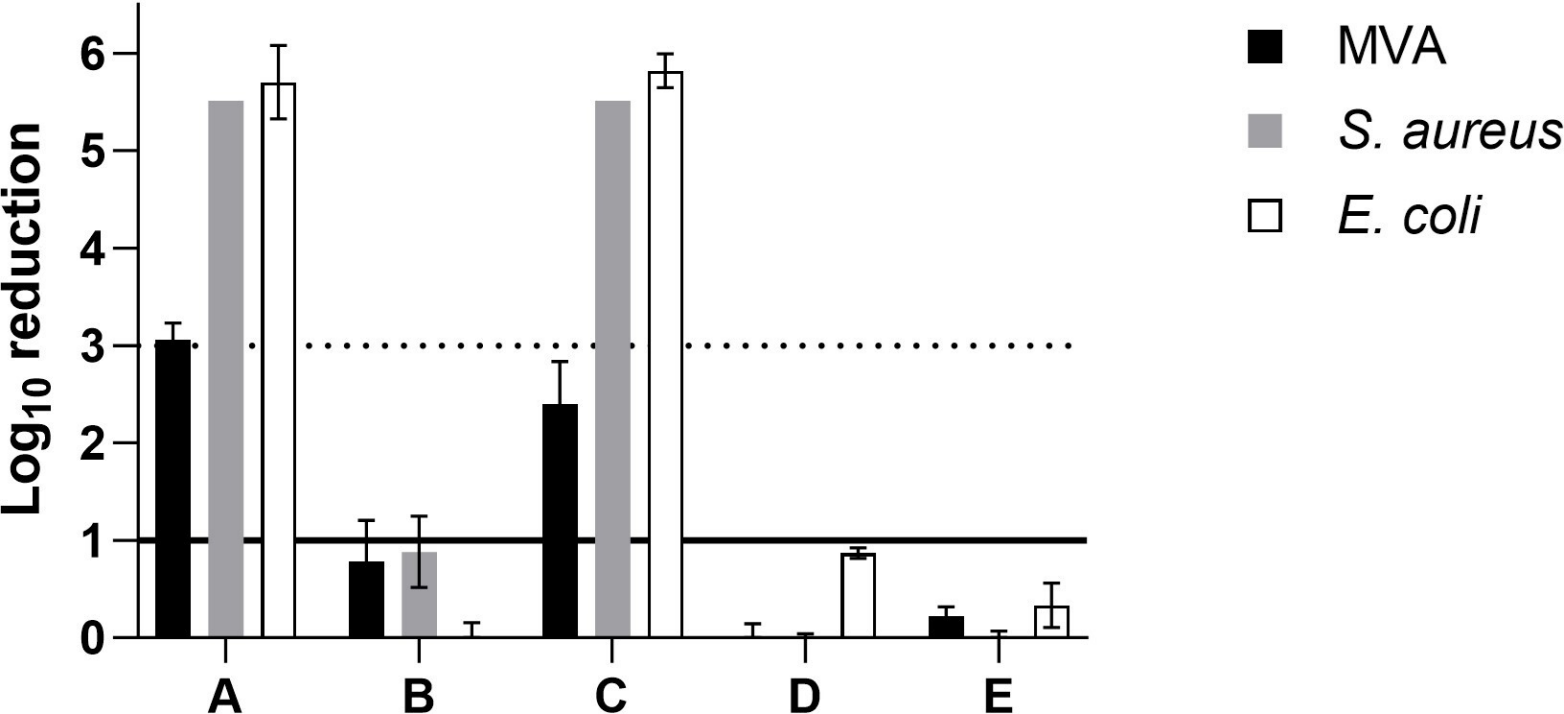
Log_10_ reduction of QAC-based coatings. *N* = 3, solid line on *y*-axis denotes significance, whereas dotted line denotes target log_10_ reduction.

Regarding QAC-based coatings (Fig. 3), samples A and C contained the same active ingredient and were the only QAC coatings that achieved more than 3 log_10_ reduction against *S. aureus* and *E. coli*, whereas for MVA, the same samples achieved 3 log_10_ reduction and 2.4 log_10_ reduction, respectively. The remaining QAC coatings showed no effect at all. Previous research revealed that QAC-containing antimicrobial products could be formulation specific. It has also been pointed out that efficacy depends not only on concentration of active ingredient, but is also influenced in general by formulation, pH of the solution, temperature, length of exposure, type of surfaces, pre-cleaning processes, and method of coating application (36–39). Although not yet fully understood, the mechanism of action of QACs is presumed to be via membrane interactions, in which the long alkyl chain permeates the cell membrane and causes membrane damage, while charged nitrogen disrupts charge distribution on cell membrane (36). Based on the results, it has been confirmed that antimicrobial activity varies with the length of the alkyl carbon chain. Our testing showed that dimethyltetradecyl[3-(trimethoxysilyl)propyl]ammonium chloride with C14 (Fig. 3A and C) has higher efficacy in comparison to dimethyloctadecyl[3-(trimethoxysilyl)propyl]ammonium chloride with C18 and alkyl(C12-16)dimethylbenzylammonium chloride (Fig. 3B, D and E). Carbon chain length C12-C16 has been shown to have greater activity and the optimum chain length against gram-negative and gram-positive bacteria are C16 and C14, respectively (36, 40, 41). Another working mechanism for immobilized QACs in coatings involves bacteria adhering to coated surfaces being subjected to lethally-strong adhesion force, resulting in membrane lipids disruption and cell death (42).

**Fig 4:**
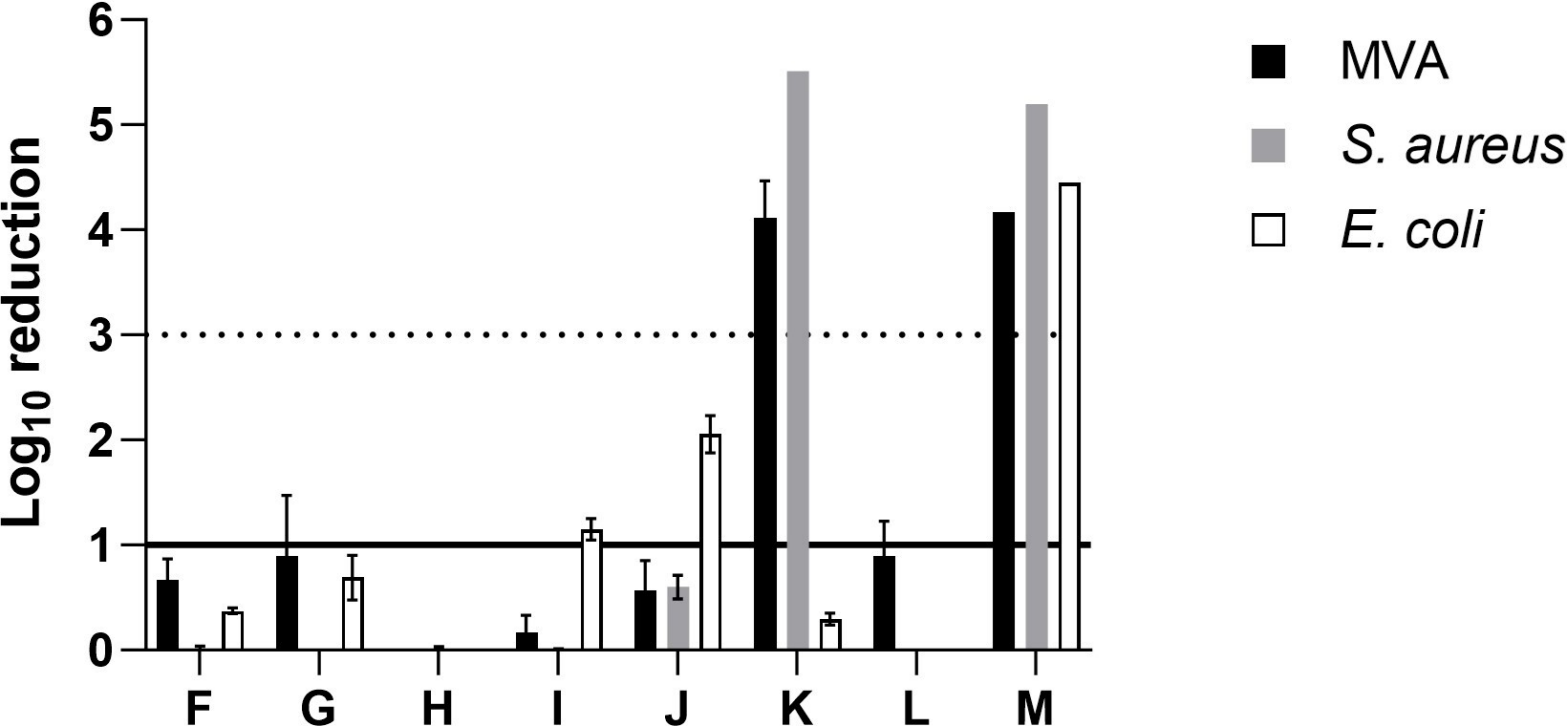
Log_10_ reduction of metal-based coatings. *N* = 3, solid line on *y*-axis denotes significance, whereas dotted line denotes target log_10_ reduction.

In terms of metal-based coating (Fig. 4), silver-based sample K was effective against MVA and *S. aureus*, achieving 4 log_10_ reduction. However, the same sample was not effective against *E. coli* (<1 log_10_ reduction), whereas silver-based sample L has no effect against all three organisms. Zinc-based samples I and J showed 1.1 log_10_ reduction and 2.1 log_10_ reduction against *E. coli*, respectively, while the remaining coating showed no antimicrobial activity. Merkl et al. showed that ZnO nanoparticle coating had no significant antiviral activity when tested against SARS-CoV-2 under a wet condition for up to 120 min at room temperature (43). Another study performed with ISO 20743 demonstrated a 3 log_10_ reduction in *S. aureus* and *E. coli* after 24 h contact time at 37°C (44). This suggests that longer exposure times may promote diffusion of active substances, thereby enhancing efficacy. Since the majority of metallic-based antimicrobial technologies such as silver, copper, zinc, and titanium rely on an ion-release mechanism (45), it can be inferred that a transport medium such as liquid is crucial for facilitating ion transport from the coated surface to microbial membrane. Some antimicrobial coatings may require moisture to be active (46, 47), and our findings confirmed the observation of a previous study that humidity has significant impact on antimicrobial activity (48). Sample M with copper-based active ingredient resulted in more than 3 log_10_ reduction against all three tested organisms. Several mechanisms of action have been reported for copper-based antimicrobial technologies, including contact-killing, ion-release, and generation of reactive oxygen species (ROS) (49). The killing efficacy of metal-based antimicrobial compounds also depends on several factors such as concentration, particle size, and the form in which these substances are presented in (ions or nanoparticles) (29). Behzadinasab et al. reported an assessment on Cu_2_O and CuO coatings against 12 microorganisms, whereas 8 out of 12 organisms were inactivated on Cu_2_O after 1 h. Besides, a 3 log_10_ reduction was observed for *P. aeruginosa* and *S. aureus* after 1 h (50). Another investigation on copper-based coating with real-life setting (drying of inoculum and short contact time) demonstrated a more than 3 log_10_ reduction antiviral effect against human coronaviruses (HCoVs) HCoV-229E and SARS-CoV-2 (51).

**Fig 5:**
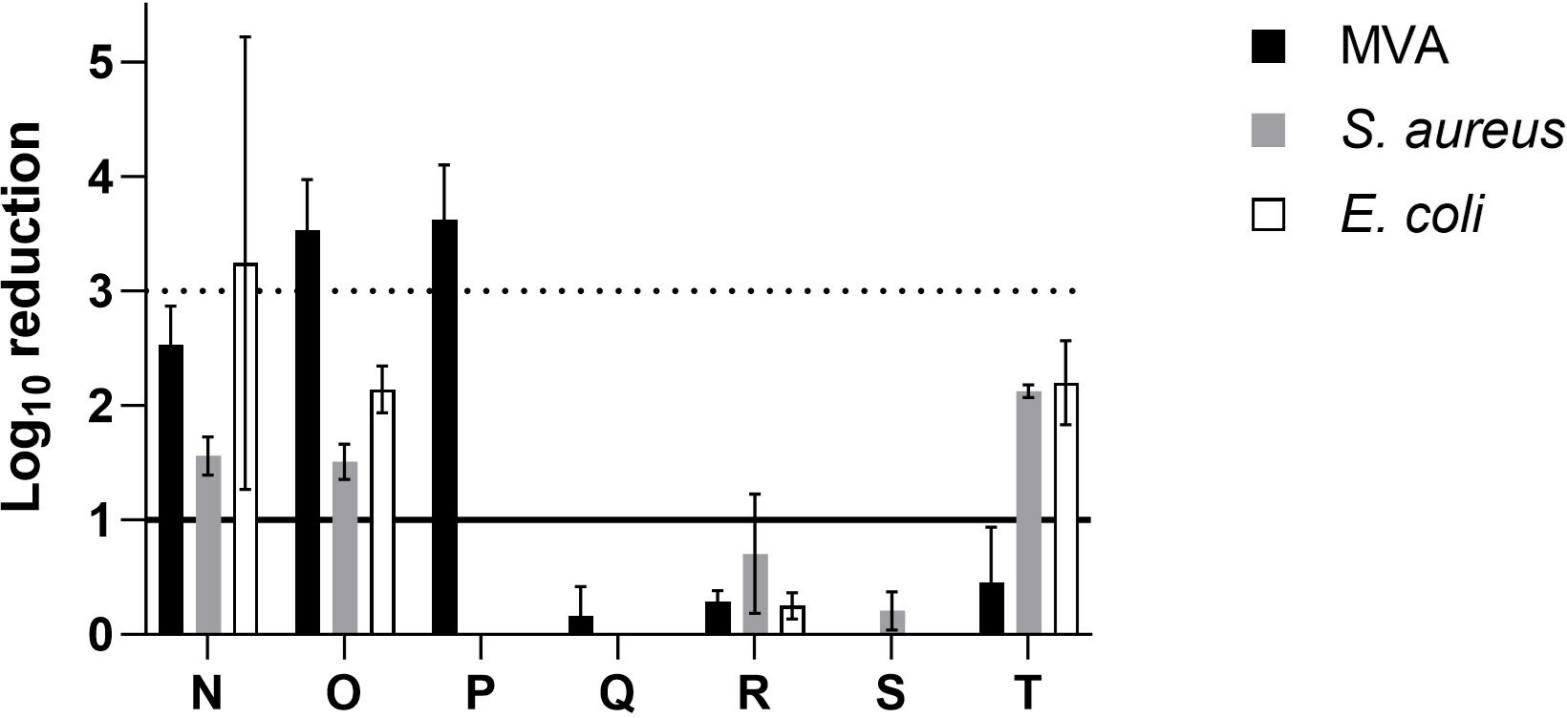
Log_10_ reduction of bio-based coatings. *N* = 3, solid line on *y*-axis denotes significance, whereas dotted line denotes target log_10_ reduction.

In the category of natural products (Fig. 5), sample N (untreated pine wood) resulted in the range of 1–2 log_10_ reduction against MVA and *S. aureus*, and 3.2 log_10_ reduction against *E. coli*. Sample O (untreated birch wood) showed 3.5 log_10_ reduction against MVA, and 1–2 log_10_ reduction against *S. aureus* and *E. coli*. Sample P (cellulose) was effective against MVA with 3.6 log_10_ reduction but showed no effect against both bacterial strains. It should be noted that microbial recovery from wooden and cellulose surfaces is complicated due to their nature (52). Important factors affecting the rate of recovery include moisture content, porosity, surface roughness, contact time, wood species, microbe species, and recovery method (52, 53). A study showed that recovery of *S. enteritidis* is lower from pine wood compared to other smooth surfaces. Therefore, it was assumed that inoculum is being absorbed and became unavailable for swab recovery (54). This also implies that bacteria retained on wood surfaces are not necessarily transferred to anything in contact with wood. Further investigation is needed to study the relationship between wooden surfaces and transmission of infection. Out of three chitosan-based samples, only sample T exhibited a moderate reduction against bacterial strains with 2.1 log_10_ reduction against *S. aureus* and 2.2 log_10_ reduction against *E. coli*. The other two samples were ineffective, likely due to the lower concentration of chitosan in samples R and S in comparison to sample T (concentration not revealed due to proprietary reasons).

Antimicrobial coatings can be classified based on their killing mechanisms such as contact-active, released-based, photoactivated, or by anti-adhesion (16). At present, combination of two or more of these mechanisms is also preferred (55). Antimicrobial coatings are prepared by solubilizing, grafting, or copolymerizing organic or inorganic biocides into conventional binders. Grafting or copolymerization process leads to formation of chemical bonds between polymer and biocide. As a result, antimicrobial coating generated with such techniques is contact-active and leaching of toxic substances into the environment can be hindered (56). Quaternary ammonium groups can be covalently grafted onto polymeric substrates. In this case, the leaching potential relies on the stability of formed chemical bond, which varies based on the selected coupling method (57). Release-based biocidal substances are commonly incorporated into polymeric matrix via physical adsorption, allowing release of biocides, such as metallic or inorganic nanoparticles, antibiotics, and so on, for inactivation of microorganisms (55). A study on antimicrobial potential of metal-coated surfaces against *S. aureus*, *E. coli*, and *L. monocytogenes* also revealed evidence of leaching when immersed in broth solutions, with copper and zinc exhibiting the highest leaching rate, while silver, titanium, iron, and molybdenum with a lower leaching rate (58). It can be deduced that the mechanism of action certainly has a significant impact on the efficacy under wet conditions. The possibility of release-based biocidal substances leaching out from the surface can lead to unrealistic results. This again highlights the drawback of wet testing method.

Furthermore, the standard testing methods aim to enhance the bioavailability of active ingredients through continuous diffusion from the surface and circulation within the thin aqueous layer of the bacterial suspension for 24 h. This prolonged exposure to leachates ultimately results in overestimated antimicrobial activity. On the other hand, under dry conditions, bacteria enter into a dormant state, which may reduce their uptake of substances from the environment.

Given the unknown effect of wet contact on microorganisms, it is crucial to consider the connection not only to drying phase that precedes the 4 h dry exposure phase during the evaluation, but also to the resuspension phase before further dilution steps. Both wet and dry contact can significantly impact the viability of bacterial species. While bacterial survival has been demonstrated in deionized water and throughout the drying and re-suspension process, critical question emerges regarding the rapid solubility and bioavailability of active biocides during the drying phase. This leads to a concern that, toward the end of the drying process, a transient surge in biocide concentration may damage the bacterial cells even before they enter the dry exposure period. Consequently, this raises the possibility that the observed effects in a study conducted over 4 h may not solely depend on the duration of dry exposure but could also be influenced by this prior damage during the earlier drying phase. To conclusively address this hypothesis, further research is necessary to examine the relationship between wet contact, the drying phase and subsequent resuspension phase. Aside from that, another crucial aspect to emphasize is the preparation of bacterial inoculum. In order to prevent further proliferation during the interim between inoculation and drying phase, bacterial test inoculum was prepared in distilled water instead of nutrient broths. Additionally, the use of physiological saline solution is precluded due its potential to impose cellular damage during the drying process. While NaCl may initially prolonged bacterial viability in the initial stage of drying process, however, as the evaporation proceeds, crystallization occurs, resulting in lethal concentration of NaCl and subsequent osmotic shock. The combined effect of desiccation and elevated NaCl concentrations results in decreased viability compared to the use of distilled water (59). This prior cell damage can again, lead to inaccuracy when assessing antimicrobial efficacy.

The screening of 20 samples revealed significantly different antimicrobial efficacy results compared to those assessed by using standard ISO test methods, showing at least two times lower log reductions. The variation in efficacy highlights the importance of testing conditions when evaluating the activity of commercial antimicrobial products. Specifically, the testing condition applied in the established methods, including a temperature of 37°C and complete submersion in fluid with 24 h contact time. Laboratory conditions often deviate from real-world settings due to differences in ambient temperature and humidity. In a typical indoor environment, the recommended operative temperature ranges between 20°C and 22°C, with a relative humidity of 30%–65% (60). Thus, the parameters used in standard testing (37°C and 24 h contact time) would be completely unrealistic for coated surfaces intended for interior use of vehicles or public transportation. Numerous researchers have also compared different testing methods and concluded that the current standard testing approach lacks relevance to dry exposure real-world conditions (18, 23, 28–30, 46, 61–63). Thus, amendment in the test method is essential for future studies, based on the mechanism of action and settings where these technologies will be applied. Since there is no universally optimal method that suits all application scenarios (air contact or liquid contact) involving different types of active substances on various surfaces, we suggest testing antimicrobial materials using the well-established conventional methods for all submerged applications. For air contact applications, we recommend employing a modified dry exposure test method, as demonstrated in this study, to prevent overestimation of antimicrobial activity. By adapting the testing approach to the specific application settings, a more accurate assessment of antimicrobial efficacy can be achieved. In this study, for instance, the test method was adjusted for use cases such as vehicle interiors, where the emphasis was on keeping the overall fomite contamination levels below a certain threshold. Therefore, an immediate disinfection effect, as needed in hospital settings, was not necessary. Additionally, it is important to note that antimicrobial surfaces should not be used as a substitute for routine cleaning procedures, as contamination is often accompanied by soiling and human skin constituents, which can potentially shield microorganisms from antimicrobial activity. For this reason, it is also worth to consider incorporating additional procedures to the test protocol in the future to better mimic real-life scenarios.

### Conclusion

Differences in experimental conditions, for instance temperature, contact time, and the form of inoculum (wet or dry) have significant impacts on antimicrobial activity. While the samples exhibit efficacy under ISO test conditions, the data presented in this study clearly indicate a lack of correlation when applying the cited methods to dry surface scenarios. Ultimately, testing methodologies should be tailored to the specific requirements, applications, and characteristics of the product or material being tested, taking into account both standardized conditions and real-world scenarios. Therefore, we suggest a revision of the standard testing method for evaluating of antimicrobial activity under dry conditions, specifically adopting materials with dry exposure scenarios as they occur especially in public and private transport settings.
